# Acute psychosis in children: do not miss immune-mediated causes

**DOI:** 10.17712/nsj.2017.1.20160552

**Published:** 2017-01

**Authors:** Mahmood D. Al-Mendalawi

**Affiliations:** *Department of Pediatrics Al-Kindy College of Medicine Baghdad University Baghdad, Iraq*

To the Editor

We have read with interest the case report by AlHakeem et al^1^ and we have 3 comments on it.

First, in their evaluation of 3 patients, the authors diagnosed immune-mediated psychosis based on the negative full work-up including metabolic screening, toxicology screening, brain magnetic resonance imaging, cerebrospinal fluid analysis, and septic screening as well as positive demonstration of some antineuronal antibodies and dramatic response to intravenous immunoglobulin (IVIG).^1^ We presume that the authors did not specifically consider systemic lupus erythematosus (SLE) as an important immune-mediated cause of psychosis. Therefore, they did not do their best to disclose it by appropriate laboratory tests. My assumption is based on the following 5 points. 1) Though no recent data are yet available on the exact national prevalence of SLE in the Kingdom of Saudi Arabia (KSA), the available data pointed out to the substantial prevalence of 19.28 per 100,000 population.^2^ 2) Neuropsychiatric picture is one of the most important manifestations of SLE. It involves a variety of clinical manifestations, including psychosis. Psychosis could be the sole and initial presentation in SLE patients. Evaluation of the clinical spectrum of pediatric SLE in KSA has shown that neuropsychiatric manifestations were seen in 30% of the cases.^3^ 3) The exact etiology of SLE remains unknown. However, a genetic component significantly contributes to the etiology of SLE. As many other autoimmune diseases, multifactorial is the most common form of inheritance. Studying the inheritance of SLE in KSA suggested the mode of inheritance as an autosomal recessive assuming Mendelian inheritance of single gene disorder.^4^ The inheritance of SLE is further strengthened by the notion that consanguineous marriage is culturally the preferred form of marriage in KSA. 4) Recently published data have shown that various autoantibodies were significantly associated with specific manifestations of neuropsychiatric disease attributed to SLE, namely, cerebrovascular events and psychosis.^5^ 5) The IVIG currently represents an important mainstay in the treatment protocol of SLE.

Second, we presume that the take-home message of the AlHakeem et al’s study^1^ includes the following: 1) Screening of the most common antineuronal antibodies related to autoimmune encephalitis/encephalopathy is needed for patients having developed a new onset psychosis. 2) In the clinical settings where facilities to detect antineuronal antibodies in patients with the first-episode psychosis are not feasible, empirical therapeutic trial with IVIG might be justifiable after exclusion of identifiable causes of psychosis has been made.

Third, patients with immune-mediated encephalitis/encephalopathy are usually seropositive for various autoantibodies directed against central nervous system neuronal surface antigens and the detection of these autoantibodies is critical to establish diagnosis. However, autoimmune panencephalitis seronegative for various autoantibodies is increasingly emerging as a subtype of autoimmune encephalitis that could present with pure neuropsychiatric features. Delay in starting immunotherapy could lead to permanent neuropsychiatric sequelae.

## Reply from the Author

We are thankful to Professor Al-Mendalawi for his comments on our article “Acute psychosis in children: do not miss immune-mediated causes”.^1^ Psychosis can be attributed to multiples etiologies, and all possible causes must be systematically examined. We agree that psychiatric manifestations, including acute psychosis, are prevalent in children with systemic lupus erythematous.^6^ However, as explained in the article text and table, systemic lupus erythematous is a well-known and well-described cause of acute psychosis. The aim of our manuscript was to increase awareness of other newly described and under-recognized immune-mediated encephalopathies/encephalitis related to other antibodies, such as N-methyl-D-aspartate receptor, leucine-rich glioma-inactivated 1, contactin-associated protein-like 2, glutamic acid decarboxylase, alpha-amino-3-hydroxy-5-methyl-4-isoxazolepropionic acid or gamma-aminobutyric acid B as a cause of acute psychosis.^7^ The 3 patients reported were screened for lupus antibodies and found negative.

We also agree and strongly recommend a full autoimmune work-up in acute psychosis, even if there are no specific findings on routine neuroimaging, electroencephalography, or cerebrospinal fluid tests. Early and aggressive immune-modulatory treatment is associated with better outcome.^7^

Brahim Tabarki

Division of Pediatric Neurology

Department of Pediatrics

Prince Sultan Military Medical City

Riyadh, Kingdom of Saudi Arabia
